# Five‐Year Trajectories of Disability in Older Adults Seeking Care for Back Pain in the Netherlands: BACE‐Dutch Cohort

**DOI:** 10.1002/ejp.70346

**Published:** 2026-08-06

**Authors:** Yanyan Fu, Trynke Hoekstra, Bart Koes, Alessandro Chiarotto

**Affiliations:** ^1^ Department of General Practice, Erasmus MC University Medical Center Rotterdam the Netherlands; ^2^ Department of Health Sciences Vrije Universiteit Amsterdam Amsterdam the Netherlands; ^3^ Amsterdam Movement Sciences, Rehabilitation & Development Amsterdam the Netherlands; ^4^ Research Unit of General Practice, Department of Public Health & Center for Muscle and Joint Health University of Southern Denmark Odense Denmark

**Keywords:** back pain, disability, older people, trajectory

## Abstract

**Background:**

Investigating trajectories in older people with back pain (BP) is informative for estimating prognosis and initiating personalized treatment. We aimed to identify 5‐year trajectories of BP‐related disability in older adults, and baseline factors associated with trajectory membership.

**Methods:**

We used data from the BACE‐Dutch cohort, which included older adults (≥ 55 years) seeking primary care for BP. Disability was measured with the Roland Morris Disability Questionnaire (RMDQ) at baseline, 6 weeks, 3, 6, 9 months and annually to 5 years. Latent class growth analysis (LCGA) identified trajectories; multinomial regression examined baseline predictors.

**Results:**

We included 669 participants. A four‐class LCGA model provided the best fit and identified four trajectories: “Recovery trajectory” (38.5%; mean baseline RMDQ = 5.4; improved), “Incomplete recovery trajectory” (28.1%; mean baseline RMDQ = 9.6; improved), “Persistent moderate disability” (22.3%; mean baseline RMDQ = 13.3; stable), and “Persistent high disability” (11.1%; mean baseline RMDQ = 17.7; stable). Most disability improvement occurred within the first 6 months, and 66.6% of participants followed improved trajectories. Older age, higher BMI, higher pain intensity, presence of depressive symptoms, less optimistic back pain beliefs, BP history, and radiating leg pain were associated with membership to worse trajectories.

**Conclusions:**

We identified four distinct 5‐year trajectories of disability in older people with BP. Most patients followed a trajectory with very low disability, while the smallest group followed a high disability trajectory. Baseline age, BMI, pain intensity, BP history, radiating leg pain, depression, and negative expectations were associated with trajectory membership.

**Significance Statement:**

In Dutch primary care, older adults with back pain show four distinct disability trajectories. Baseline characteristics associated with subgroup membership help identify patients at risk of poorer outcomes, providing input for risk stratification and a personalized care approach.

## 1 Introduction

Back pain shows a strong relationship with disability, ranking sixth among the 10 leading causes of disability‐adjusted life years (DALYs) in the age group 50–74 years in 2019 (Diseases GBD and Injuries C 2020). In older adults, the mean back pain severity shows a remarkable reduction in the first 6 weeks after presentation in primary care; however, more than 60% of them still report non‐recovery after 3 months follow‐up (Scheele et al. 2013). More than two‐thirds of people present a recurrence within 12 months after recovery (da Silva et al. 2019), and some develop a persistent and disabling condition (Gomes et al. 2023). Thus, it is important to treat back pain as a lifelong symptom rather than to focus on single episodes, which means that it is important to focus on the clinical course of back pain (Axen and Leboeuf‐Yde 2013).

Trajectory studies describing a course of repeated measures over time (Nagin 2005; Nguena Nguefack et al. 2020), explore subgroups of individuals based on their response patterns and can highlight heterogeneity in the clinical course (Nguena Nguefack et al. 2020). Previous studies have reported distinct trajectories of pain intensity and disability in patients with back pain. One Brazilian study in older patients with back pain reported three one‐year trajectories of pain intensity (‘pain recovery’, ‘incomplete pain recovery’ and ‘persistent severe pain’) and four one‐year trajectories for disability (‘disability recovery’, ‘incomplete disability recovery’, ‘persistent moderate‐severe disability’ and ‘persistent severe disability’) (Silva et al. 2022). An American study identified six one‐year pain intensity trajectories (‘moderate pain recovery’, ‘severe pain recovery’, ‘low, low moderate, moderate high and high pain’) and five one‐year disability trajectories (‘functional recovery’, ‘low, low moderate, moderate high and high disability’) in participants with low back pain (LBP) (Deyo et al. 2015). Both studies above showed the mean pain and disability scores decreased significantly in ‘recovery’ trajectories in the first 3 months while this dramatic decline was not found in other trajectories. And the prognosis was associated with biopsychosocial characteristics, like sex, BMI and comorbid conditions at baseline (Deyo et al. 2015; Silva et al. 2022). Trajectory studies are particularly relevant in older adults because this population remains under‐represented in low back pain clinical course research (Deyo et al. 2015; Paeck et al. 2014; Scheele et al. 2012; Wallwork et al. 2024) and may have distinct clinical and prognostic profiles compared with younger adults (Dionne et al. 2006; Fortin et al. 2004; Scheele et al. 2011).

In the Netherlands, a previous study (Enthoven et al. 2016) focusing on older adults with back pain (BACE‐Dutch (BACE‐D) cohort study) identified three trajectories for pain intensity over a 3‐year follow‐up period: low pain trajectory, intermediate pain trajectory and high pain trajectory. Baseline characteristics (i.e., female, high body mass index, chronic back pain, more disability, lower physical summary score of the Short‐Form 36 questionnaire and negative expectation of recovery) were associated with the membership of intermediate and high pain groups (Enthoven et al. 2016). Yet, disability, as one of the three core outcome domains in LBP (Chiarotto et al. 2016), remains underexplored in this population (Wong et al. 2019). Moreover, existing trajectory research in older adults with back pain mainly focused on a relatively short period of time (1 or 3 years). Therefore, the aims of this study were:

1. To identify trajectories for disability outcomes over a 5‐year period in older patients with back pain in the Netherlands.
2. To determine the baseline characteristics associated with the different trajectories, if any

## 2 Methods

We reported this study following the reporting guideline on latent trajectory studies, the Guidelines for Reporting on Latent Trajectory Studies (GRoLTS‐Checklist) (van de Schoot et al. 2017), see Appendix S5. A study protocol was developed before the start of the study and is provided in Appendix S7. Any deviations from the protocol are described below.

### 2.1 Study Design and Setting

We used data from the prospective BACE‐D cohort which recruited people during 2009 to September 2011, and included a follow‐up period until 2016, in the Netherlands. Full details of this cohort's design and procedures are described elsewhere (Scheele et al. 2011). The BACE‐D cohort study was approved by the Medical Ethics Committee of the Erasmus Medical Center, the Netherlands. In brief, data were collected using questionnaires, physical examination and X‐ray examination at baseline and follow‐up questionnaires at 6 weeks, 3, 6 and 9 months, and at 1, 2, 3, 4, 5 years.

### 2.2 Participants

Participants aged > 55 years old, consulting a general practitioner for a new episode of back pain, were included. A ‘new episode of back pain’ was defined as the situation in which participants had not consulted a general practitioner during the preceding 6 months for the same back complaints. All participants who could not fill in questionnaires because of language barrier or cognitive disorder, or who could not finish physical examination due to physical disability or other reasons were excluded. We would exclude participants with almost no usable baseline and follow‐up data.

### 2.3 Outcomes

Back pain related disability at baseline, 3, 6 and 9 months and at 1, 2, 3, 4 and 5 years was the primary outcome in this study. Time was modelled in months since baseline, with measurement occasions coded as 0, 3, 6, 9, 12, 24, 36, 48 and 60 months. The disability score was measured with the 24‐item Roland Morris Disability Questionnaire (RMDQ), with a score ranging from 0 to 24, with higher scores indicating worse back‐related disability (Roland and Morris 1983).

### 2.4 Statistical Analysis

We present the baseline characteristics of our study in a Table with descriptive statistics and the proportion of missing data (van de Schoot et al. 2017). To identify possible disability trajectories, we applied latent class growth analysis (LCGA) (Jung and Wickrama 2008; Nguena Nguefack et al. 2020). Disability scores measured on the 24‐item RMDQ were used in LCGA as continuous data. The process of LCGA was as follows (Jung and Wickrama 2008; Nguena Nguefack et al. 2020): **Part 1**, we hypothesized the expected number of latent classes and the shape of each trajectory over time based on background knowledge in our research field before modelling, specifically four to five trajectories for disability from studies by da Silva et al. and Deyo et al. (Deyo et al. 2015; Silva et al. 2022); **Part 2**, three‐step approach for trajectory analysis. We used *lcmm* package (Nguena Nguefack et al. 2020) for LCGA. Since statistical models like LCGA assume missing at random (MAR) (Silva et al. 2022; van de Schoot et al. 2017) and the *lcmm* estimates all models by maximum likelihood (ML). Under MAR, this approach corresponds to what is commonly referred to as “full‐information maximum likelihood (FIML)”, handling missing data on the longitudinal outcomes (Proust‐Lima VP et al. 2025; Newsom 2025; Proust‐Lima 2021). However, *lcmm* does not handle missing data of covariates (i.e., baseline variables in our case) automatically (Proust‐Lima 2021). So, in **Step 1**, we fitted LCGA without covariates and extract individuals' posterior class probabilities and class membership with the *lcmm* package in R. We tested the single‐class linear model first, then gradually added the additional classes until the maximum logical number of classes (larger than the expected number of classes based on the background knowledge, that is, 4–5 mentioned above). The *lcmm* package automatically includes participants with ≥ 1 RMDQ time point in LCGA; for more stable results or complex shapes ≥ 3 time points are recommended (4–5 time points better) (Armstrong et al. 2009; Nagin and Odgers 2010; Nylund‐Gibson and Choi 2018). Considering sample size, we used ≥ 3 time points for the main LCGA and ≥ 4 for sensitivity analysis. We used the difference in Bayesian Information Criterion (BIC) and Akaike Information Criterion (AIC) between two models with different numbers of trajectories to select the optimal number of classes; the difference in BIC and AIC was markedly reduced when the optimal number of classes was identified (Jung and Wickrama 2008; Nguena Nguefack et al. 2020; Silva et al. 2022). Another criterion of model fitness included (Enthoven et al. 2016; Nguena Nguefack et al. 2020; Silva et al. 2022) entropy (a value between 0 and 1), which indicated the separation of trajectories; values approaching 1 indicate a clear delineation of the formed trajectories. The model fit log‐likelihood indicates worse model fitness with a larger log‐likelihood value, and percentage of participants in each class (%) which was recommended more than 5% for each meaningful trajectory (Nguena Nguefack et al. 2020). The final decision on the number of classes was determined by a combination of the statistical indices mentioned above and the interpretability and clinical meaningfulness of the trajectories. Then, we tested quadric and cubic shape models. We visualized the best LCGA model through trajectory plot and reported it in a figure. **Step 2**, we reported the missingness of baseline variables and performed multiple imputation for them. **Step 3**, we extracted class membership from the best‐fitting LCGA model as the dependent variable, and baseline variables (i.e., age, sex, Body mass index (BMI), educational level, smoking, hazardous drinking, comorbidity count, comorbidity assessment (Self‐Administered Comorbidity Questionnaire [SCQ] score), back pain history, pain duration, baseline pain intensity, radiating leg pain, medication, pain catastrophizing, depression, back beliefs (The Back Beliefs Questionnaire [BBQ]), expectations of recovery) as the independent variables. Then we merged the class membership into the imputed baseline datasets, ran the multinomial logistic regression and pooled coefficients and 95% CIs via Rubin's rules. When the *p*‐value was less than 0.05, we considered the corresponding baseline variable as a predictor of class membership. **Part 3**, we then used Mplus (default FIML) to conduct the LCGA model again and compared whether model selection differs from the analysis in R. The results of the analysis in Mplus were reported as sensitivity analysis.

### 2.5 Funding Source

This research did not receive any specific grant from funding agencies in the public, commercial or not‐for‐profit sectors. The China Scholarship Council funded the first author's living expenses during the PhD period [grant numbers: 202108330084].

## 3 Results

We included 669 participants in our analysis. The BACE‐D cohort included 675 participants; we exclude six participants who only include one or two variables out of more than 500 baseline variables or participants who did not report any RMDQ score across all follow‐up time. The mean age of the study population was 66.5 (SD 7.7) years old, and the mean BMI was 27.5 kg/m^2^ (SD 4.7). Most were women (59%), non‐smokers (82%) and educated to a ‘middle’ or ‘high’ level (58%). More descriptive information is shown in Table 1. The mean RMDQ scores in the overall study population decreased most markedly from baseline (9.8 (SD 5.8)) to 3 months (7.8 (SD 6.2)), with only smaller changes thereafter (Table 2). The LCGA models analysed data from 597 participants, each with ≥ 3 RMDQ measurements over the follow‐up period. Information on the mean and variance of time within each wave is provided in Appendix S3, Table S5. Baseline characteristics according to 5‐year follow‐up status are presented in Appendix S4, Table S6.

**TABLE 1:** Descriptive analysis: Characteristics of the back pain patients at baseline (*n* = 669).

	Mean ± SD or *n* (%)	Missing value (*n*%)
Patient characteristics		
Age (years)	66.5 ± 7.7	0 (0)
Sex (female)	394 (58.9%)	0 (0)
Body mass index	27.5 ± 4.7	6 (0.9%)
Educational level		1 (0.1%)
Low	279 (41.7%)	
Middle	275 (41.1%)	
High	114 (17.0%)	
Smoking (Yes)	122 (18.2%)	2 (0.3%)
Hazardous drinking (≥ 3 in women and ≥ 4 in men)	333 (49.8%)	13 (1.9%)
Comorbidity count	2.6 ± 1.9	110 (16.4%)
Comorbidity assessment (SCQ)	4.7 ± 4.2	203 (30.3%)
Back pain characteristics		
Back pain history in last 6 months (yes)	487 (72.8%)	4 (0.6%)
Duration > 3 months	156 (23.3%)	74 (11.1%)
Back pain last week (NRS)	5.2 ± 2.7	5 (0.7%)
Radiating leg pain (yes)	379 (56.6%)	1 (0.1%)
Medication (yes)	483 (72.2%)	3 (0.4%)
Disability (RMDQ)	9.8 ± 5.8	49 (7.3%)
Psychological characteristics		
Catastrophizing (PCS)	14.1 ± 10.6	27 (4.0%)
Depression (CES‐D)	10.0 ± 7.8	51 (7.6%)
Back beliefs (BBQ)	26.4 ± 7.2	37 (5.5%)
Expectations of recovery		11 (1.6%)
Completely pain free	113 (16.9%)	
Strong improvement	178 (26.6%)	
The same as now	349 (52.2%)	
Strong worsening	15 (2.2%)	
More pain than ever	3 (0.4%)	

Abbreviations: BBQ, The Back Beliefs Questionnaire; CED‐D, Center for Epidemiologic Studies Depression Scale; NRS, Numeric Rating Scale; PCS, Pain Catastrophizing Scale; RMDQ, Roland Morris Disability Questionnaire; SCQ, Self‐Administered Comorbidity Questionnaire.

**TABLE 2:** Descriptive analysis: Follow‐up outcomes.

RMDQ sum score	Mean ± SD	Missing, *n* (%)	Minimum	Maximum	Skewness statistic
Baseline	9.8 ± 5.8	49 (7.3%)	0	23	−0.01
3‐month	7.8 ± 6.2	112 (16.7%)	0	23	0.31
6‐month	7.2 ± 6.1	126 (18.8%)	0	23	0.47
9‐month	7.2 ± 6.4	173 (25.9%)	0	23	0.45
1‐year	6.9 ± 6.4	153 (22.9%)	0	22	0.49
2‐year	7.0 ± 6.4	240 (35.9%)	0	23	0.49
3‐year	7.2 ± 6.4	291 (43.5%)	0	22	0.40
4‐year	7.0 ± 6.4	333 (49.8%)	0	22	0.49
5‐year	6.7 ± 6.2	346 (51.7%)	0	22	0.56

*Note:* RMDQ, the Roland Morris Disability Questionnaire (score range: 0–24; higher scores indicate greater disability). |Skewness Statistic| < 0.5: Typically considered approximately symmetric or only mildly skewed.

### 3.1 Trajectories

By combining the statistical indices of model fit–log‐likelihood, AIC, BIC, entropy and percentage of participants in each class (Table 3), and practical usefulness, we found the linear LCGA model with four classes outperformed other linear models with different class counts. The four‐class linear LCGA model exhibited progressively lower log‐likelihood, AIC and BIC values, excellent entropy and a reasonable distribution of participants across classes. The 4‐class cubic LCGA model performed best compared with both the four‐class linear model and the four‐class quadric models with lower log‐likelihood, AIC, and BIC values and higher entropy (Table 3). Therefore, the four‐class cubic LCGA model provided the best fit for our study population, identifying four distinct trajectories.

**TABLE 3:** Trajectory analysis: Model selection indexes of LCGA models with different classes and shape (*n* = 597)^a^.

Model	Log‐likelihood	AIC	BIC	Entropy	Percentage of participants in each class (%)				
					Class^b^ (%)	Class^c^ (%)	Class^d^ (%)	Class^e^ (%)	Class^f^
Model selection for different class									
Linear 1‐class	−13363.9	26733.9	26747.1	1	100				
Linear 2‐class	−12072.0	24156.0	24182.4	0.93	59.46	40.54			
Linear 3‐class	−11736.2	23490.4	23529.9	0.90	31.32	26.30	42.38		
**Linear 4‐class**	**−11617.2**	**23258.4**	**23311.1**	**0.88**	**22.95**	**26.63**	**39.53**	**10.89**	
Linear 5‐class	−11565.9	23161.8	23227.7	0.87	3.18	22.28	39.70	10.89	23.95
Model selection for different shape									
Linear 4‐class	−11617.2	23258.4	23311.1	0.88	22.95	26.63	39.53	10.89	
Quadric 4‐class	−11574.2	23180.3	23250.6	0.88	22.61	27.14	39.36	10.89	
**Cubic 4‐class**	**−11518.7**	**23077.4**	**23165.3**	**0.88**	**22.28**	**28.14**	**38.53**	**11.06**	

*Note:* Starting from 5‐class model, a class accounted for fewer than 5% of participants. Combining the clinical meaning of models and the indexes, we chose the 4‐class model as the best‐fit model. Bold values highlight the best‐performing models for each shape test (linear 4‐class and Cubic 4‐class).

Abbreviation: LCGA, Latent class growth analysis.

^a^ *n* = 597, LCGA models included participants with ≥ 3 valid RMDQ measurements.

^b–f^Refers to different classes, such as Class 1, Class 2, etc.

**FIGURE 1:**
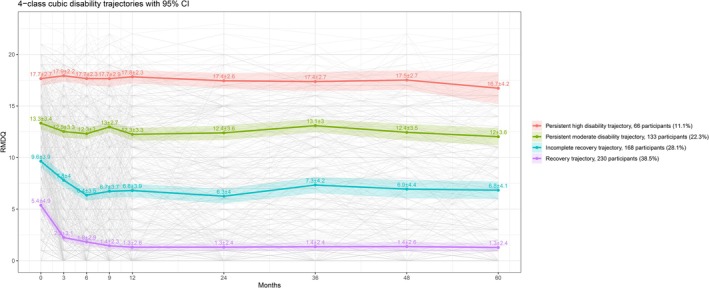
Model‐estimated four‐class cubic trajectories of disability over 60 months with 95% confidence intervals (measured using the RMDQ).

The four trajectories in our best‐fit model were defined as follows: (1) “Recovery trajectory”: 230 (38.5%) participants with a mean baseline RMDQ score of 5.4 ± 4.9 and persistently low mean scores (below 2.0) from 6 months onward; (2) “Incomplete recovery trajectory”: 168 (28.1%) participants with a mean baseline RMDQ score of 9.6 ± 3.9 and average scores between 6.0 and 7.5 after 6 months; (3) “Persistent moderate disability trajectory”: 133 (22.3%) participants with a mean baseline RMDQ score of 13.3 ± 3.4, whose mean scores remained largely unchanged over time (follow‐up means ranged from 12.0 to 13.3); (4) “Persistent high disability trajectory”: 66 (11.1%) participants with a mean baseline RMDQ score of 17.7 ± 2.7, whose follow‐up mean scores also showed only limited fluctuations (16.7–17.9), like the moderate trajectory. The improvement of disability of two favourable trajectories (“Recovery trajectory” and “Incomplete recovery trajectory”) is larger than 30% (the minimal clinically important difference [MCID]) (Ostelo et al. 2008) from baseline to 6 months. More details of the trajectories are shown in Figure 1.

### 3.2 Multinomial Regression Analysis

The “Recovery trajectory” trajectory served as the reference category in the multinomial regression analysis (Table 4). Compared to the participants in this trajectory, participants in the incomplete recovery, persistent moderate and persistent high disability trajectory were older, were female, had higher BMI, had higher baseline pain intensity and SCQ sum score, reported back pain history, radiating pain and depressive symptom, and held less optimistic beliefs about LBP prognosis. Per one‐unit increase the ORs membership of the incomplete recovery, persistent moderate and persistent high disability trajectories were 1.05/1.07/1.16 for age (years), 1.08/1.08/1.24 for BMI (kg/m^2^), 1.18/1.29/1.78 for NRS (points) and 0.95/0.89/0.89 for BBQ (points). Back pain history showed significant associations with the persistent moderate and persistent high disability trajectories: OR = 3.67 (95% CI 1.65–8.17) for persistent moderate vs. recovery disability trajectory; OR = 5.61 (95% CI 1.32–23.82) for persistent high vs. recovery disability trajectory. Radiating pain and medication were significantly associated with persistent high disability trajectories with the OR value of 5.98 (2.32–15.40) and 3.08 (1.09–8.66), respectively. Other details are showed in Table 4.

**TABLE 4:** Trajectory analysis: Multinomial logistic regression of cubic LCGA model with 4 classes (*n* = 597)^a^.

Cubic Model 4^b^	Incomplete recovery trajectory		Persistent moderate disability trajectory		Persistent high disability trajectory	
	OR (95% CI)	*p*	OR (95% CI)	*p*	OR (95% CI)	*p*
Patient characteristics						
Age (years)	**1.05 (1.02–1.09)**	**0.00**	**1.07 (1.04–1.12)**	**< 0.001**	**1.16 (1.10–1.23)**	**< 0.001**
Sex (female)	**0.61 (0.37–1.00)**	**0.05**	0.94 (0.52–1.72)	0.84	0.61 (0.25–1.45)	0.26
Body mass index (BMI)	**1.08 (1.02–1.15)**	**0.01**	**1.08 (1.01–1.17)**	**0.03**	**1.24 (1.13–1.36)**	**< 0.001**
Education level (medium vs. low)	1.65 (0.97–2.82)	0.07	1.17 (0.62–2.21)	0.63	1.06 (0.44–2.56)	0.90
Education level (high vs. low)	1.54 (0.77–3.06)	0.22	1.13 (0.46–2.75)	0.79	2.04 (0.57–7.38)	0.27
Smoking (yes)	1.12 (0.59–2.14)	0.72	1.27 (0.59–2.73)	0.53	2.45 (0.85–7.06)	0.10
Hazardous drinking (yes)	0.94 (0.59–1.51)	0.80	0.85 (0.48–1.52)	0.58	0.80 (0.35–1.83)	0.59
Comorbidity count	0.90 (0.56–1.44)	0.67	0.75 (0.44–1.26)	0.27	0.76 (0.39–1.49)	0.42
Comorbidity assessment (SCQ)	1.13 (0.87–1.46)	0.34	**1.32 (1.00–1.73)**	**0.05**	1.32 (0.96–1.82)	0.09
Pain back characteristics						
Back pain history (yes)	1.33 (0.79–2.25)	0.28	**3.67 (1.65–8.17)**	**0.00**	**5.61 (1.32–23.82)**	**0.02**
Duration > 3 months (Yes)	1.18 (0.65–2.15)	0.58	1.45 (0.74–2.86)	0.28	1.18 (0.45–3.06)	0.74
Back pain last week (NRS)	**1.18 (1.08–1.30)**	**< 0.001**	**1.29 (1.13–1.46)**	**< 0.001**	**1.78 (1.43–2.22)**	**< 0.001**
Radiating leg pain (yes)	**1.60 (1.00–2.57)**	**0.05**	1.61 (0.89–2.89)	0.11	**5.98 (2.32–15.40)**	**< 0.001**
Medication (yes)	1.43 (0.84–2.43)	0.19	1.72 (0.89–3.34)	0.11	**3.08 (1.09–8.66)**	**0.03**
Psychological characteristics						
Catastrophization (PCS)	0.98 (0.95–1.01)	0.22	1.00 (0.97–1.04)	0.99	1.03 (0.98–1.07)	0.25
Depression (CES‐D)	1.03 (0.98–1.07)	0.21	**1.07 (1.01–1.13)**	**0.01**	**1.08 (1.01–1.15)**	**0.03**
Back beliefs (BBQ)	**0.95 (0.91–0.99)**	**0.01**	**0.89 (0.85–0.94)**	**< 0.001**	**0.89 (0.83–0.96)**	**0.00**

*Note:* Bold values indicate statistically significant ORs (*p* < 0.05).

Abbreviations: BBQ, Back Beliefs Questionnaire; CES‐D, Center for Epidemiologic Studies Depression Scale; LCGA, Latent class growth analysis; NRS, numerical rating scale; PCS, Pain Catastrophizing Scale; SCQ, Self‐administered Comorbidity Questionnaire.

^a^ *n* = 597, LCGA models included participants with ≥ 3 valid RMDQ measurements.

^b^ The reference category is “Recovery trajectory”.

The results of sensitivity analyses (LCGA models included participants with ≥ 4 valid RMDQ measurements and the LCGA model constructing in Mplus) are presented in the appendices.

## 4 Discussion

This study identified four distinct disability trajectories in older patients with back pain over 5 years. Two trajectories (“Persistent moderate” and “Persistent high” disability trajectories) had high baseline RMDQ scores and remained relatively stable, with changes well below the MCID of 30%. The other two (“Recovery trajectory” and “Incomplete recovery” disability trajectories) showed marked improvement, with RMDQ scores at 5 years reduced by 74.5% and 31.6% from baseline, respectively. In the two improving trajectories, most disability improvement occurred during the first 6 months after consultation with a general practitioner, particularly in the first 3 months, after which the trajectories remained largely stable.

Previous studies have also examined disability trajectories in individuals with back pain, reporting varying numbers and characteristics (Andersen et al. 2022; Coyle et al. 2024; Deyo et al. 2015; Dutmer et al. 2020; Silva et al. 2022). Our findings closely align with those of da Silva et al. (Silva et al. 2022), who identified four trajectories in the BACE‐Brazil cohort: two with relatively stable RMDQ scores (“Persistent moderate–severe” and “Persistent severe” disability) and two with significant improvement (“Disability recovery” and “Incomplete disability recovery”). This similarity reflects the comparable inclusion criteria of both cohorts. However, in our study, the trajectory with the greatest improvement (“Recovery trajectory”) included the largest proportion of patients (round 38%), whereas in da Silva et al., the corresponding “Disability recovery” trajectory included only 13% of individuals. This difference may be caused by the lower disability levels at baseline in our cohort, as well as differences in biopsychosocial and socioeconomic characteristics between the Dutch and Brazilian populations. Methodological differences, including follow‐up duration and outcome handling, may also contribute.

Coyle et al. (Coyle et al. 2024) and Deyo et al. (Deyo et al. 2015) also examined disability trajectories in older patients with LBP. Coyle et al. identified four trajectories—two were stable, one improving and one worsening, while Deyo et al. reported five—four stable and one improving. In both studies, improvement trajectories included small number of participants (6.1% in Deyo et al., 5.7% in Coyle et al.) and had relatively high baseline disability scores. In contrast, our improvement trajectories (“Recovery disability” and “Incomplete recovery disability”) had lower baseline scores, clinically important improvement and comprised about 64% of our sample, suggesting a generally favourable prognosis. This difference likely reflects study populations: Coyle et al. included only older adults with chronic LBP, and Deyo et al. included an older sample (participants aged ≥ 65 years; mean 73.6, SD 6.7) than ours (participants aged ≥ 55 years; mean 66.5, SD 7.7). Longer pain duration is linked to poorer lower‐limb function (Reid et al. 2005); older patients whose LBP duration > 3 months show greater declines in rapid gait performance than those with shorter pain duration (Reid et al. 2005). Age is closely related to the prognosis of low back pain; higher age is associated with an increased risk of persistent pain and disability (Hayden et al. 2009).

Older adults remain under‐represented in LBP clinical course research. LBP most commonly begins between 30 and 50 years of age (Deyo et al. 2015), and this age group has been most frequently studied (Paeck et al. 2014), whereas older adults have received relatively little specific attention. A 2012 systematic review identified only five eligible studies of acute or subacute BP in populations aged ≥ 45 years and concluded that more clinical course studies in older adults were needed (Scheele et al. 2012). More than a decade later, a 2024 systematic review reached a similar conclusion: among 47 cohorts, only one specifically enrolled participants aged ≥ 60 years, and the trajectories observed in that older‐adult cohort differed from those in mixed‐age cohorts (Wallwork et al. 2024). This lack of age‐specific evidence is important because older adults differ from younger adults in clinically relevant characteristics such as multimorbidity, physical functioning and psychosocial profile. The incidence of severe BP increases with age (Dionne et al. 2006), and older adults are more likely to have one or more comorbidities than working‐age populations (Dionne et al. 2006; Fortin et al. 2004; Scheele et al. 2011). These differences may influence prognosis and patterns of disability trajectories over time. Therefore, findings from mixed‐age or younger populations may not be assumed to be generalizable to older adults, and extrapolation across age groups requires caution.

Our study found that older, female, higher BMI, higher baseline pain intensity scores, higher SCQ sum score, back pain history, radiating pain, depressive symptoms and less optimistic beliefs about LBP prognosis were significantly linked to membership of incomplete recovery, persistent moderate and persistent high disability trajectories. And compared with improving trajectories (Recovery disability trajectory), older adults having higher baseline NRS scores, back‐pain history, radiating pain and medication had much higher odds to be assigned to persistent disability trajectories. This suggests that those in persistent trajectories might be recurrent or chronic cases with more complex pain patterns, in whom symptoms are more likely to persist (da Silva et al. 2017; Traeger et al. 2021). A systematic review found that back pain history was the only consistent prognostic factor for recurrence in previous studies (da Silva et al. 2017). Higher baseline pain intensity may increase the risk of persistent symptoms (Campbell et al. 2013), and Lemmers et al. reported that patients with radiating leg pain had higher baseline disability and slower recovery (Lemmers, Melis, Pagen, et al. 2024). This finding agrees with da Silva et al. (Silva et al. 2022) and partly with other studies (Andersen et al. 2022; Coyle et al. 2024; Deyo et al. 2015; Dutmer et al. 2020; Lemmers, Melis, Pagen, et al. 2024). In addition, we also identified two psychological factors—depressive symptoms and negative beliefs about prognosis—associated with persistent moderate and persistent high disability trajectories (*p* < 0.05). This finding aligns with previous studies on disability trajectories (Coyle et al. 2024; Deyo et al. 2015; Silva et al. 2022).

### 4.1 Clinical Implications

For a primary care physician, the more clinically relevant aspects may be the patient's baseline risk profile and early clinical course. In our study, factors associated with less favourable trajectories included higher baseline disability and pain intensity, radiating leg pain, depressive symptoms, negative recovery beliefs, back pain history, higher BMI and older age. In addition, limited improvement during the first 6 months may be a clinically useful warning sign of a more persistent disability course. Although the baseline factors are not all modifiable, they may still help guide prognosis, follow‐up intensity, patient education and decisions about additional support such as psychological or multidisciplinary care.

Most older adults with back pain in Dutch primary care had a relatively favourable prognosis on disability, particularly those with lower baseline RMDQ scores and early improvement. Older adults whose baseline profile resembles the improving trajectories may be managed with education and advice to remain active. In contrast, those whose profile resembles the persistent‐disability trajectories, especially those with high baseline disability and limited early improvement, may require closer follow‐up, progressive physical exercise, psychological support or multidisciplinary care.

Among baseline variables in the multinomial model, sex and the SCQ score were associated with membership in only one subgroup. Although the effect sizes (ORs) were of moderate magnitude, the borderline *p* values render the evidence weak unlike the results in previous studies (Andersen et al. 2022; Silva et al. 2022). The significance appeared in only one trajectory make the clinical utility limited.

### 4.2 Strengths and Limitations

A key strength is the five‐year follow‐up, longer than many previous trajectory studies (Andersen et al. 2022; Coyle et al. 2024; Deyo et al. 2015; Lemmers, Melis, Hak, et al. 2024; Lemmers, Melis, Pagen, et al. 2024; Silva et al. 2022). We also ran two sensitivity analyses to test robustness to missingness and software choice. However, the percentage of missing data increased significantly after 1 year, reaching 51.7% at the five‐year follow‐up; we cannot completely rule out the possibility of attrition bias, particularly if missingness was related to unobserved outcomes.

## 5 Conclusions

Our study identified four disability trajectories over 5 years including two stable trajectories with relatively high baseline RMDQ scores and two trajectories with marked disability improvement. More than half of participants had a good prognosis on back pain‐related disability. Baseline characteristics like age, BMI, baseline NRS scores, back pain history, radiating leg pain, depression, and negative expectation were associated with membership of disability trajectories.

## Author Contributions

This study was designed by Y.F., B.K. and A.C. The main data analysis was performed by Y.F. and the sensitivity analysis was performed by Y.F. and T.H. The results were critically examined by all authors. Y.F. had a primary role in preparing the manuscript, which was edited by Y.F., T.H., B.K. and A.C. All authors have approved the final version of the manuscript and agree to be accountable for all aspects of the work.

## Funding

This work was funded by China Scholarship Council (CSC) scholarships.

## Conflicts of Interest

The authors declare no conflicts of interest.

## Supporting information


**Appendix S1:** ejp70346‐sup‐0001‐Supinfo1.docx.
**Appendix S2:** ejp70346‐sup‐0001‐Supinfo1.docx.
**Appendix S3:** ejp70346‐sup‐0001‐Supinfo1.docx.
**Appendix S4:** ejp70346‐sup‐0001‐Supinfo1.docx.
**Appendix S5:** ejp70346‐sup‐0001‐Supinfo1.docx.
**Appendix S6:** ejp70346‐sup‐0001‐Supinfo1.docx.
**Appendix S7:** ejp70346‐sup‐0001‐Supinfo1.docx.

**Data S1:** ejp70346‐sup‐0002‐Supinfo2.pdf.

**Data S2:** ejp70346‐sup‐0003‐Supinfo3.doc.
