# Supplementary material for: Five‐Year Trajectories of Disability in Older Adults Seeking Care for Back Pain in the Netherlands: BACE‐Dutch Cohort
Source: Eur J Pain. 2026 Aug 6;30(7):e70346. doi: 10.1002/ejp.70346 (PMC13444210; doi:10.1002/ejp.70346)
Supplement: Supplementary file 1 [file EJP-30-0-s003.pdf]

```
### Load dataset-----
 ### read the incomplete dataset including all the variables we need
MIdata<-read.csv("/Users/fuyanyan/Library/CloudStorage/OneDrive-
ErasmusMC/20251107test/data/analysis/incom.analysis data.csv", colClasses = c(patientcode
= "character")) ###do not let R recognize the patientcode as a predictor
 ##indicate the variable type in this data frame
library(dplyr)
continuous_vars <- c("ARDQ_totaal", "DRDQ_totaal", "ERDQ_totaal", "FRDQ_totaal",
"GRDQ_totaal", "HRDQ_totaal", "JRDQ_totaal", "KRDQ_totaal", "LRDQ_totaal",
                     "Age", "BMI",
"comorbidity_count","comorbidity_sumscore","NRSlastweek", "APCS_totaal", "ABBQ_tot",
"ACES_tot","days_3m", "days_6m", "days_9m", "days_1y",
                     "days_2y", "days_3y", "days_4y", "days_5y")
categorical_vars <- c("Sex", "EducationLevel", "Smoke", "Drinking_risk", "BPhistory",
"chronic", "radiating_pain", "Medication","AExp1")
MIdata <- MIdata %>% mutate(across(all_of(continuous_vars), as.numeric)) %>%
mutate(across(all_of(categorical_vars), as.factor))
MIdata <- MIdata %>% mutate(Sex = factor(Sex, levels = c(1, 2),labels = c("Male",
"Female")),
                            EducationLevel = factor(EducationLevel,levels = 1:3, labels =
c("low", "medium", "high")),
                            Smoke = factor(Smoke,levels = c(0, 1), labels = c("No",
"Yes")),
                            Drinking_risk = factor(Drinking_risk, levels = c(0, 1), labels
= c("No", "Yes")),
                            BPhistory = factor(BPhistory, levels = c(0, 1), labels =
c("No", "Yes")),
                            chronic = factor(chronic, levels = c(0, 1), labels = c("No",
"Yes")),
                            radiating_pain = factor(radiating_pain, levels = c(0, 1),
labels = c("No", "Yes")),
                            Medication = factor(Medication, levels = c(0, 1),labels =
c("No", "Yes")),
                            AExp1=factor(AExp1, levels = 1:5,labels = c("Completely pain
free", "Strong improvement", "The same as now", "Strong worsening", "More pain than
ever"),ordered = TRUE))
str(MIdata)
write.csv(MIdata, "MIdata_allbaseline.csv", row.names = FALSE) ### save the dataset fits R

## exclude extreme follow-up time values based on deviation from scheduled time -----
time_vars <- c("days_3m", "days_6m", "days_9m", "days_1y","days_2y", "days_3y", "days_4y",
"days_5y")
wave_labels <- c("3 months", "6 months", "9 months", "1 year","2 years", "3 years", "4
years", "5 years")
planned_days <- c(days_3m = 90,
                  days_6m = 180,
                  days_9m = 270,
                  days_1y = 365,
                  days_2y = 730,
                  days_3y = 1095,
                  days_4y = 1460,
                  days_5y = 1825)

cutoffs <- c(days_3m = 30,
             days_6m = 30,
             days_9m = 30,
             days_1y = 60,
             days_2y = 60,
             days_3y = 90,
             days_4y = 90,
             days_5y = 90)
#a copy of the data for summarizing after excluding extreme values
MIdata_trim <- MIdata
#Set negative numbers or values with excessively large deviations to NA
for (v in time_vars) {
  diff_from_plan <- MIdata_trim[[v]] - planned_days[v]
  MIdata_trim[[v]][MIdata_trim[[v]] < 0 | abs(diff_from_plan) > cutoffs[v]] <- NA}
time_table_trim <- data.frame(
  Wave = wave_labels,
  n = sapply(MIdata_trim[time_vars], function(x) sum(!is.na(x))),
  `Mean days since baseline` = round(sapply(MIdata_trim[time_vars], function(x) mean(x,
```

```
na.rm = TRUE)), 1),
  SD = round(sapply(MIdata_trim[time_vars], function(x) sd(x, na.rm = TRUE)), 1),
  Variance = round(sapply(MIdata_trim[time_vars], function(x) var(x, na.rm = TRUE)), 1),
  Min = sapply(MIdata_trim[time_vars], function(x) min(x, na.rm = TRUE)),
  Max = sapply(MIdata_trim[time_vars], function(x) max(x, na.rm = TRUE)),
  check.names = FALSE)
time_table_trim
write.csv(time_table_trim, "time_within_wave_summary_trimmed.csv", row.names = FALSE)

## compare baseline characteristics by 5-year follow-up status -----
MIdata$miss_5y <- ifelse(is.na(MIdata$LRDQ_totaal), 1, 0)
MIdata$miss_5y <- factor(
  MIdata$miss_5y,
  levels = c(0, 1),
  labels = c("Completed 5-year follow-up", "Missing 5-year follow-up"))
table(MIdata$miss_5y, useNA = "ifany")
continuous_baseline <- c("Age", "BMI", "comorbidity_count",
"comorbidity_sumscore","NRSlastweek", "ARDQ_totaal","APCS_totaal", "ACES_tot", "ABBQ_tot")
categorical_baseline <- c("Sex", "EducationLevel", "Smoke", "Drinking_risk","BPhistory",
"chronic", "radiating_pain","Medication", "AExp1")
#continuous variable comparison
cont_results_5y <- lapply(continuous_baseline, function(v) {
  x0 <- MIdata[MIdata$miss_5y == "Completed 5-year follow-up", v, drop = TRUE]
  x1 <- MIdata[MIdata$miss_5y == "Missing 5-year follow-up", v, drop = TRUE]
  test <- t.test(x0, x1)
  data.frame(
    Variable = v,
    `Completed 5-year follow-up` = sprintf(
      "%.1f (%.1f)",mean(x0, na.rm = TRUE), sd(x0, na.rm = TRUE)),
    `Missing 5-year follow-up` = sprintf(
      "%.1f (%.1f)", mean(x1, na.rm = TRUE), sd(x1, na.rm = TRUE)),
    p_value = ifelse(test$p.value < 0.001, "<0.001", sprintf("%.3f", test$p.value)),
    stringsAsFactors = FALSE)})
cont_results_5y <- do.call(rbind, cont_results_5y)
cont_results_5y
write.csv(cont_results_5y, "baseline_continuous_by_5y_followup.csv", row.names = FALSE)
#categorical variable comparison
cat_results_5y <- lapply(categorical_baseline, function(v) {
  tab <- table(MIdata[[v]], MIdata$miss_5y, useNA = "no")
  test <- chisq.test(tab)
  complete_n <- colSums(tab)["Completed 5-year follow-up"]
  missing_n  <- colSums(tab)["Missing 5-year follow-up"]
  rows <- lapply(rownames(tab), function(level) {
    n0 <- tab[level, "Completed 5-year follow-up"]
    n1 <- tab[level, "Missing 5-year follow-up"]
    data.frame(
      Variable = v,
      Level = level,
      `Completed 5-year follow-up` = sprintf("%d (%.1f%%)", n0, 100 * n0 / complete_n),
      `Missing 5-year follow-up` = sprintf("%d (%.1f%%)", n1, 100 * n1 / missing_n),
      p_value = ifelse(test$p.value < 0.001, "<0.001", sprintf("%.3f", test$p.value)),
      stringsAsFactors = FALSE)})
  do.call(rbind, rows)})
cat_results_5y <- do.call(rbind, cat_results_5y)
cat_results_5y
write.csv(cat_results_5y, "baseline_categorical_by_5y_followup.csv", row.names = FALSE)

###To test the number of patients whose valid observations of RMDQ >= 1,2,3,and 4-----
outcomes <- c("ARDQ_totaal","DRDQ_totaal","ERDQ_totaal","FRDQ_totaal",
              "GRDQ_totaal","HRDQ_totaal","JRDQ_totaal","KRDQ_totaal","LRDQ_totaal")
num_cov  <- c("Age","BMI","comorbidity_count","comorbidity_sumscore",
              "NRSlastweek","APCS_totaal","ABBQ_tot","ACES_tot")
bin_cov  <- c("Sex","Smoke","BPhistory","chronic","radiating_pain","Medication",
"Drinking_risk")
multi_cov<- c("EducationLevel")
ord_cov  <- c("AExp1")

library(dplyr)
obs_n <- rowSums(!is.na(MIdata[ , outcomes]))
sum_1 <- sum(obs_n >= 1)
sum_2 <- sum(obs_n >= 2)
sum_3 <- sum(obs_n >= 3)
```

```
sum_4 <- sum(obs_n >= 4)
prop_1 <- mean(obs_n >= 1)
prop_2 <- mean(obs_n >= 2)
prop_3 <- mean(obs_n >= 3)
prop_4 <- mean(obs_n >= 4)

MIdata$obs_n <- rowSums(!is.na(MIdata[, outcomes]))
MIdata_sub2 <- subset(MIdata, obs_n >= 2)
MIdata_sub3 <- subset(MIdata, obs_n >= 3)
MIdata_sub4 <- subset(MIdata, obs_n >= 4)

###LCGA-----
 ##transfer wide format to long format for analysis datasets
library(tidyr)
months   <- c(0, 3, 6, 9, 12, 24, 36, 48, 60)
time_map <- setNames(months, outcomes)

 ##for the original dataset, which only excluded the case without any RMDQ score, like the
Mplus.
MIdata_long <- MIdata%>%
  pivot_longer(all_of(outcomes), names_to = "follow_up", values_to = "RDQ") %>%
  mutate(Time = unname(time_map[follow_up])) %>%
  arrange(patientcode, Time)
 #Preprocessing, generate a stable numerical ID for each patientcode and retain the
mapping
key_id <- MIdata_long %>%
  distinct(patientcode) %>%
  arrange(patientcode) %>%
  mutate(IDnum = as.integer(factor(patientcode, levels = patientcode)))
library(lcmm)
dat <- MIdata_long %>%
  filter(!is.na(RDQ)) %>%
  left_join(key_id, by = "patientcode") %>%
  arrange(IDnum, Time)
  id_var <- "IDnum"
 ##fit one class as the starting point
m1_lin  <- hlme(fixed = RDQ ~ Time, subject = id_var,ng = 1, data = dat, verbose = FALSE)
m2_lin <- gridsearch(rep = 50, maxiter = 10, minit = m1_lin, m=hlme(RDQ ~ Time, subject =
id_var, ng = 2, data = dat, mixture = ~ Time, verbose = FALSE))
m3_lin <- gridsearch(rep = 50, maxiter = 10, minit = m1_lin, m=hlme(RDQ ~ Time, subject =
id_var, ng = 3, data = dat, mixture = ~ Time, verbose = FALSE))
m4_lin <- gridsearch(rep = 50, maxiter = 10, minit = m1_lin, m=hlme(RDQ ~ Time, subject =
id_var, ng = 4, data = dat, mixture = ~ Time, verbose = FALSE))
m5_lin <- gridsearch(rep = 100, maxiter = 10, minit = m1_lin, m=hlme(RDQ ~ Time, subject =
id_var, ng = 5, data = dat, mixture = ~ Time, verbose = FALSE))
m6_lin <- gridsearch(rep = 100, maxiter = 10, minit = m1_lin, m=hlme(RDQ ~ Time, subject =
id_var, ng = 6, data = dat, mixture = ~ Time, verbose = FALSE))
summarytable(m1_lin, m2_lin, m3_lin, m4_lin, m5_lin, m6_lin, which =
c("G","loglik","conv","npm","AIC","BIC","entropy","%class"))

 ##the main analysis with cases having at least 3 RMDQ scores-----
MIdata_sub3_long <- MIdata_sub3 %>%
  pivot_longer(all_of(outcomes), names_to = "follow_up", values_to = "RDQ") %>%
  mutate(Time3 = unname(time_map[follow_up])) %>%
  arrange(patientcode, Time3)
 #Preprocessing, generate a stable numerical ID for each patientcode and retain the
mapping
key_id3 <- MIdata_sub3_long %>%
  distinct(patientcode) %>%
  arrange(patientcode) %>%
  mutate(IDnum3 = as.integer(factor(patientcode, levels = patientcode)))
library(lcmm)
dat3 <- MIdata_sub3_long %>%
  filter(!is.na(RDQ)) %>%
  left_join(key_id3, by = "patientcode") %>%
  arrange(IDnum3, Time3)
id_var3 <- "IDnum3"
 #fit one class as the starting point
m1_lin3  <- hlme(fixed = RDQ ~ Time3, subject = id_var3,ng = 1, data = dat3, verbose =
FALSE)
m2_lin3 <- gridsearch(rep = 50, maxiter = 10, minit = m1_lin3, hlme(RDQ ~ Time3, subject =
id_var3, ng = 2, data = dat3, mixture = ~ Time3, verbose = FALSE))
```

```
m3_lin3 <- gridsearch(rep = 50, maxiter = 10, minit = m1_lin3, hlme(RDQ ~ Time3, subject =
id_var3, ng = 3, data = dat3, mixture = ~ Time3, verbose = FALSE))
m4_lin3 <- gridsearch(rep = 50, maxiter = 10, minit = m1_lin3, hlme(RDQ ~ Time3, subject =
id_var3, ng = 4, data = dat3, mixture = ~ Time3, verbose = FALSE))
m5_lin3 <- gridsearch(rep = 100, maxiter = 10, minit = m1_lin3, hlme(RDQ ~ Time3, subject
= id_var3, ng = 5, data = dat3, mixture = ~ Time3, verbose = FALSE))
m6_lin3 <- gridsearch(rep = 100, maxiter = 10, minit = m1_lin3, hlme(RDQ ~ Time3, subject
= id_var3, ng = 6, data = dat3, mixture = ~ Time3, verbose = FALSE))
summarytable(m1_lin3, m2_lin3, m3_lin3, m4_lin3, m5_lin3, m6_lin3, which =
c("G","loglik","conv","npm","AIC","BIC","entropy","%class"))
 #trajectory shape test
m1_quad3 <- hlme(fixed = RDQ ~ poly(Time3, 2, raw = TRUE), subject = id_var3, ng = 1, data
= dat3, verbose = FALSE)
m4_quad3 <- gridsearch(rep = 50, maxiter = 50, minit = m1_quad3, hlme(RDQ ~ poly(Time3, 2,
raw = TRUE), subject = id_var3, ng = 4, data = dat3, mixture = ~ poly(Time3, 2, raw =
TRUE), verbose = FALSE))
summarytable(m4_quad3, which =
c("G","loglik","conv","npm","AIC","BIC","entropy","%class"))
m1_cub3  <- hlme(fixed = RDQ ~ poly(Time3, 3, raw = TRUE), subject = id_var3,ng = 1, data
= dat3, verbose = FALSE)
m4_cub3  <- gridsearch(rep = 50, maxiter = 50, minit = m1_cub3, hlme(RDQ ~ poly(Time3, 3,
raw = TRUE), subject = id_var3, ng = 4, data = dat3, mixture = ~ poly(Time3, 3, raw =
TRUE), verbose = FALSE))
summarytable(m4_cub3, which = c("G","loglik","conv","npm","AIC","BIC","entropy","%class"))

 ##the sensitivity analysis with cases having at least 4 RMDQ scores
MIdata_sub4_long <- MIdata_sub4 %>%
  pivot_longer(all_of(outcomes), names_to = "follow_up", values_to = "RDQ") %>%
  mutate(Time4 = unname(time_map[follow_up])) %>%
  arrange(patientcode, Time4)
 #Preprocessing, generate a stable numerical ID for each patientcode and retain the
mapping
key_id4 <- MIdata_sub4_long %>%
  distinct(patientcode) %>%
  arrange(patientcode) %>%
  mutate(IDnum4 = as.integer(factor(patientcode, levels = patientcode)))
dat4 <- MIdata_sub4_long %>%
  filter(!is.na(RDQ)) %>%
  left_join(key_id4, by = "patientcode") %>%
  arrange(IDnum4, Time4)
id_var4 <- "IDnum4"
 #fit one class as the starting point
m1_lin4  <- hlme(fixed = RDQ ~ Time4, subject = id_var4,ng = 1, data = dat4, verbose =
FALSE)
m2_lin4 <- gridsearch(rep = 50, maxiter = 10, minit = m1_lin4, hlme(RDQ ~ Time4, subject =
id_var4, ng = 2, data = dat4, mixture = ~ Time4, verbose = FALSE))
m3_lin4 <- gridsearch(rep = 50, maxiter = 10, minit = m1_lin4, hlme(RDQ ~ Time4, subject =
id_var4, ng = 3, data = dat4, mixture = ~ Time4, verbose = FALSE))
m4_lin4 <- gridsearch(rep = 50, maxiter = 10, minit = m1_lin4, hlme(RDQ ~ Time4, subject =
id_var4, ng = 4, data = dat4, mixture = ~ Time4, verbose = FALSE))
m5_lin4 <- gridsearch(rep = 100, maxiter = 10, minit = m1_lin4, hlme(RDQ ~ Time4, subject
= id_var4, ng = 5, data = dat4, mixture = ~ Time4, verbose = FALSE))
m6_lin4 <- gridsearch(rep = 100, maxiter = 10, minit = m1_lin4, hlme(RDQ ~ Time4, subject
= id_var4, ng = 6, data = dat4, mixture = ~ Time4, verbose = FALSE))
summarytable(m1_lin4, m2_lin4, m3_lin4, m4_lin4, m5_lin4, m6_lin4, which =
c("G","loglik","conv","npm","AIC","BIC","entropy","%class"))
 #trajectory shape test
m1_quad4 <- hlme(fixed = RDQ ~ poly(Time4, 2, raw = TRUE), subject = id_var4, ng = 1, data
= dat4, verbose = FALSE)
m4_quad4 <- gridsearch(rep = 50, maxiter = 50, minit = m1_quad4, hlme(RDQ ~ poly(Time4, 2,
raw = TRUE), subject = id_var4, ng = 4, data = dat4, mixture = ~ poly(Time4, 2, raw =
TRUE), verbose = FALSE))
summarytable(m4_quad4, which =
c("G","loglik","conv","npm","AIC","BIC","entropy","%class"))
m1_cub4  <- hlme(fixed = RDQ ~ poly(Time4, 3, raw = TRUE), subject = id_var4,ng = 1, data
= dat4, verbose = FALSE)
m4_cub4  <- gridsearch(rep = 50, maxiter = 50, minit = m1_cub4, hlme(RDQ ~ poly(Time4, 3,
raw = TRUE), subject = id_var4, ng = 4, data = dat4, mixture = ~ poly(Time4, 3, raw =
TRUE), verbose = FALSE))
summarytable(m4_cub4, which = c("G","loglik","conv","npm","AIC","BIC","entropy","%class"))

 ##trajectory plots for main analysis-----
```

```r
library(dplyr)
library(ggplot2)
library(tidyr)   # for pivot_longer
library(lcmm)
 plot_lcga_model <- function(mod,
                            dat,
                            id_name = "IDnum3",
                            time_name = "Time3",
                            outcome_name = "RDQ",
                            model_title = "Trajectory plot",
                            class_descriptions = NULL) {

  # 1) Extract class membership
  pp_df <- extract_classes(mod, dat, id_name)

  dat_tmp <- dat %>%
    dplyr::left_join(pp_df, by = id_name) %>%
    dplyr::mutate(Class = factor(class))

  # If 1-class model, make sure Class exists nicely
  if (nlevels(dat_tmp$Class) == 0 || all(is.na(dat_tmp$Class))) {
    dat_tmp$Class <- factor("1")}

  # 2) Compute observed mean / SD / SE / 95% CI by class x time
  obs_stats <- dat_tmp %>%
    group_by(Class, .data[[time_name]]) %>%
    summarise(
      mean_obs = mean(.data[[outcome_name]], na.rm = TRUE),
      sd_obs   = sd(.data[[outcome_name]], na.rm = TRUE),
      n        = sum(!is.na(.data[[outcome_name]])),
      .groups  = "drop"
    ) %>%
    mutate(
      se_obs = sd_obs / sqrt(pmax(n, 1)),
      lwr    = mean_obs - 1.96 * se_obs,
      upr    = mean_obs + 1.96 * se_obs)

  # 3) Order classes by baseline mean, descending
  baseline_time <- min(obs_stats[[time_name]], na.rm = TRUE)
  lvl <- obs_stats %>%
    filter(.data[[time_name]] == baseline_time) %>%
    arrange(desc(mean_obs)) %>%
    pull(Class) %>%
    unique()
  obs_stats <- obs_stats %>%
    mutate(Class = factor(Class, levels = lvl))
  dat_tmp <- dat_tmp %>%
    mutate(Class = factor(Class, levels = lvl))

  # 4) Build legend labels
  class_counts <- dat_tmp %>%
    distinct(.data[[id_name]], Class) %>%
    count(Class, name = "n")
  total_n <- sum(class_counts$n)
  # default descriptions if not supplied
  if (is.null(class_descriptions)) {
    default_pool <- c(
      "Persistent high disability trajectory",
      "Persistent moderate disability trajectory",
      "Incomplete recovery trajectory",
      "Recovery trajectory",
      "Class 5 trajectory",
      "Class 6 trajectory"
    )
    class_descriptions <- default_pool[seq_along(lvl)]
  } else {
    class_descriptions <- class_descriptions[seq_along(lvl)]
  }
  legend_df <- tibble(
    Class = factor(lvl, levels = lvl),
    desc  = class_descriptions
  ) %>%
```

```
    left_join(class_counts, by = "Class") %>%
    mutate(
      pct = 100 * n / total_n,
      legend_lab = sprintf("%s, %d participants (%.1f%%)", desc, n, pct))

  # 5) Plot
  p <- ggplot() +
    geom_line(
      data = dat_tmp,
      aes(x = .data[[time_name]], y = .data[[outcome_name]], group = .data[[id_name]]),
      colour = "grey80", alpha = 0.25, linewidth = 0.3
    ) +
    geom_ribbon(
      data = obs_stats,
      aes(x = .data[[time_name]], ymin = lwr, ymax = upr, fill = Class),
      alpha = 0.20, colour = NA
    ) +
    geom_line(
      data = obs_stats,
      aes(x = .data[[time_name]], y = mean_obs, colour = Class),
      linewidth = 1.1
    ) +
    geom_point(
      data = obs_stats,
      aes(x = .data[[time_name]], y = mean_obs, colour = Class),
      size = 1.6
    ) +
    geom_text(
      data = obs_stats,
      aes(
        x = .data[[time_name]],
        y = mean_obs,
        label = paste0(round(mean_obs, 1), "±", round(sd_obs, 1)),
        colour = Class
      ),
      vjust = -0.6, size = 3, show.legend = FALSE
    ) +
    scale_x_continuous(breaks = c(0, 3, 6, 9, 12, 24, 36, 48, 60)) +
    labs(
      x = "Months",
      y = "RMDQ",
      title = model_title
    ) +
    scale_colour_discrete(
      breaks = legend_df$Class,
      labels = legend_df$legend_lab
    ) +
    scale_fill_discrete(
      breaks = legend_df$Class,
      labels = legend_df$legend_lab
    ) +
    theme_bw() +
    theme(
      legend.position = "right",
      legend.title = element_blank()
    )
  return(p)}
#for mi_lin3 model
p_m1_lin3 <- plot_lcga_model(
  mod = m1_lin3,
  dat = dat3,
  model_title = "1-class linear disability trajectory"
)
p_m1_lin3
#for m2_lin3 model
p_m2_lin3 <- plot_lcga_model(
  mod = m2_lin3,
  dat = dat3,
  model_title = "2-class linear disability trajectories",
  class_descriptions = c(
    "Higher disability trajectory",
    "Lower disability trajectory"))
```

```r
  p_m2_lin3
  #for m3_lin3 model
  p_m3_lin3 <- plot_lcga_model(
    mod = m3_lin3,
    dat = dat3,
    model_title = "3-class linear disability trajectories",
    class_descriptions = c(
      "High disability trajectory",
      "Moderate disability trajectory",
      "Low disability trajectory"))
  p_m3_lin3
  #for m4_lin3 model
  p_m4_lin3 <- plot_lcga_model(
    mod = m4_lin3,
    dat = dat3,
    model_title = "4-class linear disability trajectories",
    class_descriptions = c(
      "Persistent high disability trajectory",
      "Persistent moderate disability trajectory",
      "Incomplete recovery trajectory",
      "Recovery trajectory"))
  p_m4_lin3
  #for m5_lin3 model
  p_m5_lin3 <- plot_lcga_model(
    mod = m5_lin3,
    dat = dat3,
    model_title = "5-class linear disability trajectories",
    class_descriptions = c(
      "Highest disability trajectory",
      "High disability trajectory",
      "Moderate disability trajectory",
      "Low disability trajectory",
      "Lowest disability trajectory"))
  p_m5_lin3
  #for m6_lin3 model
  p_m6_lin3 <- plot_lcga_model(
    mod = m6_lin3,
    dat = dat3,
    model_title = "6-class linear disability trajectories",
    class_descriptions = c(
      "Class 1 trajectory",
      "Class 2 trajectory",
      "Class 3 trajectory",
      "Class 4 trajectory",
      "Class 5 trajectory",
      "Class 6 trajectory"))
  p_m6_lin3
  #for m4_quad3 model
  p_m4_quad3 <- plot_lcga_model(
    mod = m4_quad3,
    dat = dat3,
    model_title = "4-class quadratic disability trajectories",
    class_descriptions = c(
      "Persistent high disability trajectory",
      "Persistent moderate disability trajectory",
      "Incomplete recovery trajectory",
      "Recovery trajectory"))
  p_m4_quad3
  #for m4_cubic3 model
  p_m4_cub3 <- plot_lcga_model(
    mod = m4_cub3,
    dat = dat3,
    model_title = "4-class cubic disability trajectories with 95% CI",
    class_descriptions = c(
      "Persistent high disability trajectory",
      "Persistent moderate disability trajectory",
      "Incomplete recovery trajectory",
      "Recovery trajectory"))
  p_m4_cub3

 ##prepare for multinomial regression------
library(dplyr)
```

```
library(tidyr)
final_fit <- m4_cub3
# extract the posterior probability by postprob()
extract_pprob <- function(fit, dat = NULL){
  x <- try(lcmm::postprob(fit), silent = TRUE)
  if (!inherits(x, "try-error")) {
    if (is.list(x) && "pprob" %in% names(x) && is.data.frame(x$pprob)) return(x$pprob)
    if (is.data.frame(x) && ("class" %in% names(x) || any(grepl("^prob", names(x)))))
return(x)
    if (is.list(x)) {
      dfs <- Filter(is.data.frame, x)
      for (df in dfs) {
        if ("class" %in% names(df) || any(grepl("^prob", names(df)))) return(df)
      }
    }
  }
  if (!is.null(dat)) {
    x2 <- try(lcmm::postprob(fit, data = dat), silent = TRUE)
    if (!inherits(x2, "try-error")) {
      if (is.list(x2) && "pprob" %in% names(x2) && is.data.frame(x2$pprob))
return(x2$pprob)
      if (is.data.frame(x2) && ("class" %in% names(x2) || any(grepl("^prob", names(x2)))))
return(x2)
      if (is.list(x2)) {
        dfs <- Filter(is.data.frame, x2)
        for (df in dfs) {
          if ("class" %in% names(df) || any(grepl("^prob", names(df)))) return(df)
        }
      }
    }
  }
  if (!is.null(fit$pprob)) return(as.data.frame(fit$pprob))
  stop("Cannot find posterior-probabilities data frame.")
}
pp <- extract_pprob(final_fit, dat = dat3)
library(dplyr)
library(rlang)
pp <- as.data.frame(pp)
 #make the ID Integer type for generate and merge new variables
id_col <- intersect(c("IDnum3","subject","ID","id"), names(pp))[1]
if (is.na(id_col)) stop("No ID column found in 'pp'.")
# if 'class' isn't there yet, derive it from prob* columns (safeguard)
if (!("class" %in% names(pp))) {
  prob_cols <- grep("^prob", names(pp), value = TRUE)
  if (length(prob_cols) < 2) stop("No 'class' column and not enough prob* columns in
'pp'.")
  pp$class <- max.col(as.matrix(pp[, prob_cols, drop = FALSE]), ties.method = "first")
}
pp <- pp %>%
  dplyr::rename(IDnum3 = !!rlang::sym(id_col)) %>%     # unquote string column name
  dplyr::mutate(IDnum3 = as.integer(IDnum3),
                ClassML = paste0("Class ", class)) %>%
  dplyr::select(IDnum3, ClassML, class, dplyr::starts_with("prob"))
#match the ID with patientcode and based on that merge the new class variables into the
wide dataset
pp <- as.data.frame(pp)
pp <- pp %>% dplyr::distinct(IDnum3, .keep_all = TRUE)
class_info <- key_id3 %>%
  dplyr::select(patientcode, IDnum3) %>%
  dplyr::inner_join(pp %>% dplyr::select(IDnum3, class, ClassML), by = "IDnum3")
reg_df <- MIdata_sub3 %>%               # wide dataset (obs_n >= 3)
  dplyr::left_join(class_info, by = "patientcode")
View(reg_df)
 #recode the class and ClassML
library(dplyr)
reg_df <- reg_df %>%
  dplyr::mutate(
    class = dplyr::recode(as.integer(class),
                          `1` = 2L, `2` = 4L, `3` = 1L, `4` = 3L,
                          .default = NA_integer_),
    class = factor(class)
  ) %>%
```

```
  dplyr::mutate(
    ClassML = dplyr::recode(as.character(ClassML),
                             "Class 1" = "Low",
                             "Class 2" = "High",
                             "Class 3" = "Very low",
                             "Class 4" = "Moderate",
                             .default = NA_character_),
    ClassML = factor(ClassML))
 #check the number of each class with the plot
table(reg_df$class, useNA = "ifany")
table(reg_df$ClassML, useNA = "ifany")

### multiple imputation and multinomial regression------
str(reg_df)
library(mice)      # multiple imputation
library(nnet)      # multinomial regression
library(MASS)      # polr() used by mice for ordered factors
 ##select the related variable to make a new dataframe for MI
id_varmi <- "patientcode"
y_varmi  <- "class"
num_covmi   <- c("Age","BMI","comorbidity_count","comorbidity_sumscore",
               "NRSlastweek","APCS_totaal","ABBQ_tot","ACES_tot")
bin_covmi   <-
c("Sex","Smoke","BPhistory","chronic","radiating_pain","Medication","Drinking_risk")
multi_covmi <- c("EducationLevel")
ord_covmi   <- c("AExp1")
mi_df <- reg_df %>% dplyr::select(all_of(id_varmi), all_of(y_varmi),all_of(num_covmi),
all_of(bin_covmi), all_of(multi_covmi), all_of(ord_covmi))
 ##starting MI
ini  <- mice(mi_df, maxit = 0, printFlag = FALSE)
meth <- ini$method
pred <- ini$predictorMatrix
 #Do NOT impute ID and class, and do NOT use them as predictors
meth[id_varmi] <- ""
meth[y_varmi]  <- ""
pred[, id_varmi] <- 0
pred[, y_varmi]  <- 0
pred[id_varmi, ] <- 0
pred[y_varmi, ]  <- 0
 #ran MI
set.seed(20251107)
imp <- mice(mi_df, m = 20, maxit = 20, method = meth, predictorMatrix = pred, printFlag =
FALSE)
baseline_vars <- c(num_covmi, bin_covmi, multi_covmi, ord_covmi)
check_vars    <- c(baseline_vars, y_varmi)
datscheckmi   <- complete(imp, action = "all")  # list of m data frames for check if the
imputation is complete
na_mat <- sapply(datscheckmi, function(d) colSums(is.na(d[, check_vars, drop = FALSE])))
na_mat[ rowSums(na_mat)>0 , , drop=FALSE ]
any(na_mat > 0) #if it is the FALSE, then the imputation is complete.
all_complete <- sapply(datscheckmi, function(d) all(!is.na(d[, check_vars, drop =
FALSE])))
all_complete #if it is true then the imputation is complete

 ##have a look of trace plot for imputation
 #choose the variables have big percentage of missing
plot(imp,
c("comorbidity_count","comorbidity_sumscore","chronic","APCS_totaal","ABBQ_tot","ACES_tot"))
stripplot(imp, comorbidity_count + comorbidity_sumscore + chronic + APCS_totaal + ABBQ_tot
+ ACES_tot ~ .imp,
          pch = 16, cex = .6, jitter = TRUE)
library(lattice)
densityplot(imp, ~ comorbidity_count + comorbidity_sumscore + chronic + APCS_totaal +
ABBQ_tot + ACES_tot, layout = c(3, 2))

 ##multinomial refgression
imp$data$class <- factor(imp$data$class, levels = 1:4, ordered = FALSE)
imp$data$class <- relevel(imp$data$class, ref = "1")
imp$data <- within(imp$data, {
  Sex            <- relevel(Sex, ref = "Male")
  Smoke          <- relevel(Smoke, ref = "No")
  BPhistory      <- relevel(BPhistory, ref = "No")
```

```
  chronic        <- relevel(chronic, ref = "No")
  radiating_pain <- relevel(radiating_pain, ref = "No")
  Medication     <- relevel(Medication, ref = "No")
  Drinking_risk  <- relevel(Drinking_risk, ref = "No")
  EducationLevel <- relevel(EducationLevel, ref = "low")})
imp$data$AExp1_ord <- factor(imp$data$AExp1,
                             levels = c("Completely pain free","Strong improvement",
                                        "The same as now","Strong worsening","More pain
than ever"),
                             ordered = TRUE)
imp$data$AExp1_num <- as.numeric(imp$data$AExp1_ord)
library(nnet)
fit_mn <- with(
  imp,
  nnet::multinom(
    class ~ Age + BMI + comorbidity_count + comorbidity_sumscore +
      NRSlastweek + APCS_totaal + ABBQ_tot + ACES_tot +
      Sex + Smoke + BPhistory + chronic + radiating_pain + Medication +
      Drinking_risk + EducationLevel + AExp1_num,
    trace = FALSE
  )
)
pool_mn <- pool(fit_mn)
res <- summary(pool_mn, conf.int = TRUE)
if (!"conf.low"  %in% names(res)) res$conf.low  <- res$`2.5 %`
if (!"conf.high" %in% names(res)) res$conf.high <- res$`97.5 %`
# 2) If 'outcome' column is missing, reconstruct it from the first fitted model
if (!"outcome" %in% names(res)) {
  mod1 <- fit_mn$analyses[[1]]                # first multinom fit
  # In multinom(), the reference is the first level of the factor
  nonref_levels <- mod1$lev[-1]               # e.g., "2","3","4"
  # Estimate how many terms per outcome block in the summary
  block_size <- nrow(res) / length(nonref_levels)
  if (abs(block_size - round(block_size)) > .Machine$double.eps^0.5) {
    # fallback: use unique term count as block size
    block_size <- length(unique(res$term))
  } else {
    block_size <- round(block_size)
  }
  outcome_vec <- rep(nonref_levels, each = block_size)
  # Trim or recycle to match rows just in case
  if (length(outcome_vec) != nrow(res)) {
    outcome_vec <- rep(nonref_levels, length.out = nrow(res))
  }
  res$outcome <- outcome_vec
}
# 3) Build a clean OR table with readable outcome labels and export
lab <- setNames(paste0("Class ", res$outcome, " vs 1"), unique(res$outcome))
res_or <- res %>%
  dplyr::filter(term != "(Intercept)") %>%
  dplyr::mutate(
    outcome = if ("outcome" %in% names(res))
      factor(outcome, levels = c("2","3","4"),
             labels = c("Class 2 vs 1","Class 3 vs 1","Class 4 vs 1"))
    else factor("Contrast", levels = "Contrast"),
    OR  = exp(estimate),
    LCI = exp(conf.low),
    UCI = exp(conf.high),
    `OR (95% CI)` = sprintf("%.2f (%.2f–%.2f)", OR, LCI, UCI),
    p = ifelse(p.value < 0.001, "<0.001", sprintf("%.3f", p.value))
  ) %>%
  dplyr::select(outcome, term, OR, LCI, UCI, `OR (95% CI)`, p)
library(tibble)
res_or <- as_tibble(res_or)
print(res_or, n = Inf)
write.csv(res_or, "multinom_OR_95CI_by_outcome.csv", row.names = FALSE)
```
