# Supplementary material for: Five‐Year Trajectories of Disability in Older Adults Seeking Care for Back Pain in the Netherlands: BACE‐Dutch Cohort
Source: Eur J Pain. 2026 Aug 6;30(7):e70346. doi: 10.1002/ejp.70346 (PMC13444210; doi:10.1002/ejp.70346)
Supplement: Supplementary file 2 [file EJP-30-0-s001.docx]

**Appendix 1. Sensitivity analysis with participants including at least 4 time points**

**Table 1s. Model selection indexes of linear LCGA models with participants including at least 4 time points (n=563)***

| **Model^a^** | **Class(es)** | **Log-likelihood** | **BIC** | **AIC** | **Entropy** | **Percentage of participants in each class (%)** | | | | |
| --- | --- | --- | --- | --- | --- | --- | --- | --- | --- | --- |
|  |  |  |  |  |  | **Class1** | **Class2** | **Class3** | **Class4** | **Class5** |
| Model 1 | 1 | -13016.2 | 26051.42 | 26038.4 | 1.00 | 100.00 |  |  |  |  |
| Model 2 | 2 | -11757.1 | 23552.3 | 23526.3 | 0.93 | 39.96 | 60.04 |  |  |  |
| Model 3 | 3 | -11429.3 | 22915.5 | 22876.5 | 0.90 | 25.40 | 31.79 | 42.81 |  |  |
| **Model 4** | **4** | **-11312.6** | **22701.1** | **22649.1** | **0.88** | **39.96** | **27.00** | **9.95** | **23.09** |  |
| Model 5 | 5 | -11261.2 | 22617.4 | 22552.4 | 0.88 | 22.56 | 24.16 | 9.95 | 40.14 | 3.20 |

a. Combine all the indexes in Table 1s and the clinical meaning reported by previous studies, we finally choose the model 4 as our best-fit linear model. Then we tested the shape of model 4 with quadratic and cubic transformation.

*n=563, LCGA models included participants with ≥4 valid RMDQ measurements.

**Table 2s. Model selection indexes of 4-classes LCGA models participants including at least 4 time points (n=563) ***

| **Model^b^** | **Class(es)** | **Log-likelihood** | **BIC** | **AIC** | **Entropy** | **Percentage of participants in each class (%)** | | | |
| --- | --- | --- | --- | --- | --- | --- | --- | --- | --- |
|  |  |  |  |  |  | **Class1** | **Class2** | **Class3** | **Class4** |
| Linear model 4 | 4 | -11312.6 | 22701.1 | 22649.1 | 0.88 | 39.96 | 27.00 | 9.95 | 23.09 |
| Quadratic model 4 | 4 | -11271.3 | 22643.8 | 22574.5 | 0.89 | 39.79 | 27.53 | 9.95 | 22.74 |
| Cubic model 4 | 4 | -11217.6 | 22561.8 | 22475.1 | 0.89 | 39.08 | 28.42 | 9.95 | 22.56 |

b. Combine all the indexes in Table 2s, we finally choose the Cubic model 4 as our best-fit linear model.
c. *n=563, LCGA models included participants with ≥4 valid RMDQ measurements.

**Appendix 2. Sensitivity analysis in Mplus**

**Table 3s. Model selection indexes of linear LCGA models with different classes in Mplus with FIML (n=662) ***

| **Model^a^** | **log likelihood** | **AIC** | **BIC** | **Entropy** | **Percentage of participants in each class (%)** | | | | |
| --- | --- | --- | --- | --- | --- | --- | --- | --- | --- |
|  |  |  |  |  | **Class1** | **Class2** | **Class3** | **Class4** | **Class5** |
| 1-class model | -13,680 | 27,382,067 | 27,431,515 | 1.00 | 100.00 |  |  |  |  |
| 2-class model | -12,348 | 24,725,789 | 24,788,723 | 0.91 | 42.45 | 57.55 |  |  |  |
| 3-class model | -11,977 | 23,988,286 | 24,064,706 | 0.87 | 29.60 | 42.15 | 28.25 |  |  |
| 4-class model | -11,837 | 23,714,010 | 23,803,915 | 0.85 | 23.72 | 11.78 | 38.22 | 26.28 |  |
| 5-class model | -11,772 | 23,590,638 | 23,694,029 | 0.85 | 3.02 | 23.56 | 22.96 | 38.67 | 11.78 |

a. Combine all the indexes in Table 1s and the clinical meaning reported by previous studies, we finally choose the model 4 as our best-fit linear model. Then we tested the shape of model 4 with quadratic and cubic transformation.

*n=662, by default, Mplus LCGA models include participants with ≥1 valid RMDQ measurement.

**Table 4s. Model selection indexes of LCGA models with 4 classes in different shape in Mplus with FIML (n=662) ***

| **Model^b^** | **Class(es)** | **Log-likelihood** | **BIC** | **AIC** | **Entropy** | **Percentage of participants in each class (%)** | | | |
| --- | --- | --- | --- | --- | --- | --- | --- | --- | --- |
|  |  |  |  |  |  | **Class1** | **Class2** | **Class3** | **Class4** |
| Linear model 4 | 4 | -11,837 | 23,714,010 | 23,803,915 | 0.85 | 23.72 | 11.78 | 38.22 | 26.28 |
| Quadratic model 4 | 4 | -11,811,952 | 23,671,903 | 23,779,790 | 0.851 | 23.72 | 11.93 | 38.07 | 26.28 |
| Cubic model 4 | 4 | -11,764,257 | 23,584,513 | 23,710,381 | 0.851 | 23.41 | 27.04 | 37.61 | 11.93 |

b. Combine all the indexes in Table 2s, we finally choose the Cubic model 4 as our best-fit linear model.

*n=662, by default, Mplus LCGA models include participants with ≥1 valid RMDQ measurement.

**Appendix 3.**

**Table 5s Descriptive statistics of follow-up time within each wave ***

| Wave | n | Mean days since baseline | SD | Variance | Min | Max |
| --- | --- | --- | --- | --- | --- | --- |
| 3 months | 578 | 94.2 | 7.5 | 56.5 | 60 | 120 |
| 6 months | 539 | 187.0 | 7.5 | 56.7 | 152 | 210 |
| 9 months | 505 | 277.5 | 7.4 | 54.6 | 241 | 300 |
| 1 year | 542 | 370.0 | 11.4 | 130.0 | 308 | 425 |
| 2 years | 443 | 736.3 | 11.2 | 126.2 | 685 | 788 |
| 3 years | 410 | 1103.8 | 15.6 | 243.0 | 1061 | 1184 |
| 4 years | 361 | 1467.2 | 12.9 | 167.0 | 1429 | 1535 |
| 5 years | 354 | 1834.1 | 17.3 | 300.3 | 1760 | 1908 |

* Cases with negative follow-up day and cases with substantial deviations from the scheduled follow-up time were excluded. Substantial deviation was defined as >30 days for the 3-, 6-, and 9-month waves, >60 days for the 1- and 2-year waves, and >90 days for the 3-, 4-, and 5-year waves.

**Appendix 4.**

**Table 6s. Baseline characteristics according to 5-year follow-up status**

| Variable |  | Completed 5-year-follow-up | Missing 5-year-follow-up | P-value |
| --- | --- | --- | --- | --- |
| Continuous variable |  |  |  |  |
|  | Age | 65.7 (6.8) | 67.2 (8.4) | 0.01 |
|  | BMI | 27.2 (4.4) | 27.7 (4.9) | 0.17 |
|  | Comorbidity count | 2.4 (1.9) | 2.8 (1.9) | 0.01 |
|  | Comorbidity assessment (SCQ) | 4.1 (3.8) | 5.3 (4.4) | 0.00 |
|  | Back pain last week (NRS) | 5.1 (2.7) | 5.2 (2.7) | 0.48 |
|  | Disability (RMDQ) | 9.4 (5.9) | 10.1 (5.8) | 0.15 |
|  | Catastrophizing (PCS) | 13.8 (10.6) | 14.4 (10.6) | 0.50 |
|  | Depression (CES-D) | 9.4 (7.8) | 10.6 (7.7) | 0.05 |
|  | back beliefs (BBQ) | 27.0 (7.3) | 25.9 (7.1) | 0.05 |
| Categorical variable |  |  |  |  |
|  | Sex (Female) | 141 (43.7%) | 134 (38.7%) | 0.22 |
|  | Education level |  |  |  |
|  | Low | 112 (34.7%) | 167 (48.4%) | 0.00 |
|  | Medium | 145 (44.9%) | 130 (37.7%) | 0.00 |
|  | High | 66 (20.4%) | 48 (13.9%) | 0.00 |
|  | Smoking (Yes) | 49 (15.2%) | 73 (21.2%) | 0.06 |
|  | Hazardous drinking (≥3 in women and ≥4 in men) | 179 (55.9%) | 154 (45.8%) | 0.01 |
|  | Back pain history in last 6 months (Yes) | 224 (69.6%) | 263 (76.7%) | 0.05 |
|  | Duration > 3 months | 67 (22.7%) | 89 (29.7%) | 0.07 |
|  | Radiating leg pain (Yes) | 184 (57.0%) | 195 (56.5%) | 0.97 |
|  | Medication (Yes) | 247 (76.7%) | 236 (68.6%) | 0.02 |
|  | Expectations of recovery |  |  |  |
|  | Completely pain free | 68 (21.5%) | 45 (13.2%) | 0.05 |
|  | Strong improvement | 80 (25.2%) | 98 (28.7%) | 0.05 |
|  | The same as now | 162 (51.1%) | 187 (54.8%) | 0.05 |
|  | Strong worsening | 5 (1.6%) | 10 (2.9%) | 0.05 |
|  | More pain than ever | 2 (0.6%) | 1 (0.3%) | 0.05 |

SCQ, Self-Administered Comorbidity Questionnaire

NRS, Numeric Rating Scale

RMDQ, Roland Morris Disability Questionnaire

PCS, Pain Catastrophizing Scale

CED-D, Center for Epidemiologic Studies Depression Scale

BBQ, The Back Beliefs Questionnaire

**Appendix 5. GRoLTS checklist**

| Checklist Item | Reported? | Details |
| --- | --- | --- |
| 1. Is the metric of time used in the statistical model reported? | Yes | In Figure 1, time was modelled in months since baseline, with measurement occasions coded as 0, 3, 6, 9, 12, 24, 36, 48, and 60 months. We added a statement in the Methods (page 2, line 92-93). |
| 2. Is information presented about the mean and variance of time within a wave? | Yes* | In appendix 3, Table 5s. We added a statement in Results (page 4, line152). |
| 3a. Is the missing data mechanism reported? | Yes | Page 3, line 104-106. |
| 3b. Is a description provided of what variables are related to attrition/missing data? | Yes* | In appendix 4, Table 6s. We added a statement in Results (page 4, line153). |
| 3c. Is a description provided of how missing data in the analyses were dealt with? | Yes | Page 3, Statistical analysis section. |
| 4. Is information about the distribution of the observed variables included? | Yes | Table 2. |
| 5. Is the software mentioned? | Yes | Page 3 line 104 and 134-136. |
| 6a. Are alternative specifications of within-class heterogeneity considered (e.g., LGCA vs. LGMM) and clearly documented? If not, wassufficient justification provided as to eliminate certain specifications from consideration? | No | Our research question was to explore whether there are heterogeneous subgroups in long-term disability outcomes within the BACE population. The LCGA model was sufficient to answer this question. In addition, we aimed to use the same method as a previous BACE-D trajectory study on pain outcomes. |
| 6b. Are alternative specifications of the between-class differences in variance–covariance matrix structure considered and clearly documented? If not, was sufficient justification provided as to eliminate certain specifications from consideration? | No | Based on 6a, we did not test alternative variance–covariance structures because we used LCGA, with growth factor variances fixed to zero. |
| 7. Are alternative shape/functional forms of the trajectories described? | Yes | Page 3, Statistical analysis section. |
| 8. If covariates have been used, can analyses still be replicated? | Yes | Page 3, Statistical analysis section. |
| 9. Is information reported about the number of random start values and final iterations included? | Yes | In our R code, for the final model, 50 random starts with a maximum of 50 iterations were used in the grid search. We upload the R code document as a supplementary material. |
| 10. Are the model comparison (and selection) tools described from a statistical perspective? | Yes | Page 3, Statistical analysis section. |
| 11. Are the total number of fitted models reported, including a one-class solution? | Yes | Page 3, Statistical analysis section, and Table 3. |
| 12. Are the number of cases per class reported for each model (absolute sample size, or proportion)? | Yes | Table 3. |
| 13. If classification of cases in a trajectory is the goal, is entropy reported? | Yes | Table 3. |
| 14a. Is a plot included with the estimated mean trajectories of the final solution? | Yes | Figure 1. |
| 14b. Are plots included with the estimated mean trajectories for each model? | Yes | Appendix 6. |
| 14c. Is a plot included of the combination of estimated means of the final model and the observed individual trajectories split out for each latent class？ | Yes | Figure 1 and Appendix 6, Colored thick lines: estimated mean trajectories for each latent class.  Grey thin lines: observed individual RMDQ trajectories. |
| 15. Are characteristics of the final class solution numerically described (i.e., means, SD/SE, n, CI, etc.)? | Yes | This information is presented across Table 3, Table 4, and Figure 1. |
| 16. Are the syntax files available (either in the appendix, supplementary materials, or from the authors)? | Yes | We upload the R code document as a supplementary material. |

* Not assessed in the initial analysis; added during revision.

**Appendix 6. Trajectory plots of each model in main analysis**

**Appendix 7. Protocol of this research project**

**Five-year trajectories of physical functioning in older adults seeking care for back pain**

**Introduction**

Back pain is one of the most common symptoms causing a large burden on health care systems worldwide from teenage to old age [1]. In addition, back pain shows a strong relationship with disability, ranking sixth among the ten leading causes of disability-adjusted life years (DALYs) in the age group 50-74 years in 2019 [1]. According to Scheele et.al., in older adults the mean back pain severity shows a remarkable reduction in the first 6 weeks, however, more than 60% of them still report non-recovery after 3 months follow-up [2]. Moreover, recurrence is common in back pain, in fact, more than two-thirds of people present a recurrence within 12 months after recovery [3], and some develop a persistent and disabling condition [4]. Thus, it is important to treat back pain as a lifelong condition rather than to focus on single episodes, which means that it is important to focus on the clinical course of back pain [5].

A clinical trajectory describes a course of repeated measures over time [6, 7]. Trajectories explore the relationships among individuals and aim to find different subgroups or classes based on personal response patterns [7]. A previous study reviewing various trajectories on low back pain found that it is necessary to investigate trajectories in different age groups for treatment implications because older adults are more likely not to respond to back pain treatment [8]. Trajectories of patients in different counties with varying characteristics can provide us a more comprehensive view of the back pain course and help us understand the heterogeneity in the clinical course. One Brazilian study in older patients with back pain reported three one-year trajectories in pain intensity (‘pain recovery’, ‘incomplete pain recovery’ and ‘persistent severe pain’) and four one-year trajectories in physical functioning (‘disability recovery’, ‘incomplete disability recovery’, ‘persistent moderate-severe disability’ and ‘persistent severe disability’) [9]. An American study identified six one-year pain intensity trajectories ('moderate pain recovery’, ‘severe pain recovery’, ‘low, low moderate, moderate high and high pain’) and five one-year physical functioning trajectories (‘functional recovery’, ‘low, low moderate, moderate high and high disability’) in participants with LBP [10]. Generally, both studies above showed the mean pain and physical functioning scores decreased significantly in ‘recovery' trajectories in the first 3 months while this dramatic decline was not found in other trajectories, and the prognosis of pain intensity and physical functioning was associated with some of biopsychosocial characteristics, like sex, BMI, comorbid conditions at baseline [9, 10]. In the Netherlands, a previous study [11] focusing on older adults with back pain (BACE-Dutch (BACE-D) cohort study) identified three trajectories for pain intensity over a 3-year follow-up period: low pain trajectory, intermediate pain trajectory and high pain trajectory. Additionally, the authors found that several baseline characteristics (i.e. female, high body mass index, chronic back pain, more disability, lower physical summary score of SF-36, and negative expectation of recovery) were associated with the membership of intermediate and high pain groups [11]. Nevertheless, existing trajectory research in older patients with back pain mainly focused on a relatively short period of time (1 or 3 years). And, in the previous study based on the BACE-D cohort, the authors focused only on pain intensity as an outcome. In fact, another important outcome that needs to be evaluated in this patient population is physical functioning [12]. Therefore, the aims of this study are:

1. To identify distinct clinical trajectories for physical functioning outcomes over a 5-year period in older patients with back pain in Netherlands;
2. To determine the baseline characteristics associated with the different trajectories.

**Method**

We will report this study following the reporting guideline on latent trajectory studies, the GRoLTS-Checklist [13].

*Study design and setting*

We will use data from the prospective BACE-D cohort which recruited people during 2009 to September 2011, and kept follow-up until 2016, in the Netherlands. Full details of this cohort’s design and procedures are described elsewhere [14]. In brief, data was collected using questionnaires, physical examination, and X-ray examination at baseline and follow-up questionnaires at 6 weeks, 3, 6, and 9 months, and at 1, 2, 3, 4, 5 years. The BACE-D cohort study was approved by the Medical Ethics Committee of the Erasmus Medical Center, the Netherlands.

*Participants*

Participants aged > 55 years old, consulting a general practitioner for a new episode of back pain, were included. A ‘new episode of back pain’ was defined as the situation in which participants had not consulted a general practitioner during the preceding 6 months for the same back complaints. All participants who were not able to fill in questionnaires because of language barrier or cognitive disorder, or who could not finish physical examination due to physical disability or other reasons were excluded.

*Outcomes*

Back pain related physical functioning at baseline, 3, 6 and 9 months, and at 1, 2, 3, 4 and 5 years is the primary outcomes in this study. Physical functioning score was measured with the 24-item Roland Morris Disability Questionnaire (RMDQ), with a score ranging from 0 to 24, with higher scores indicating worse back-related disability [15].

*Statistical analysis*

We will use descriptive statistics for the sample baseline characteristics. To identify possible physical functioning trajectories, latent class growth analysis (LCGA) will be used [7, 16]. Physical functioning scores measured on the 24-item RMDQ will be used in LCGA as continuous data. The process of LCGA is as follows [7, 16]: ***Step 1a,*** we will hypothesize the expected number of latent classes and the shape of each trajectory over time based on background knowledge in our research field before modelling, specifically four to five trajectories for disability from studies by da Silva et al. and Deyo et al. [9, 10]. ***Step 1b,*** to better predict the number of latent classes and the shape of trajectories during LCGA modeling, we will draw a spaghetti plot in SPSS to describe individual trajectories of each patient (i.e. figure 1). ***Step 2***, we will specify a single-class linear and a quadratic latent growth curve model first, that is a simple univariate latent growth curve formed by our outcome variables repeatedly measured at different follow-up time points. Then we will gradually add the additional classes until the maximum logical number of classes (larger than the expected number of classes based on the background knowledge, i.e. 4 to 5 mentioned above). We will use the difference in Bayesian Information Criterion (BIC) between two models with different numbers of trajectories to select the optimal number of classes; the difference in BIC will be markedly reduced when the optimal number of classes is identified [7, 9, 16]. For example, we can initially test the LCGA models from a three-class model, then test LCGA models with four, five and six classes. Among these models, the best fitting model will have the smallest BIC value. Other criterion of model fitness includes [7, 9, 11]: entropy (a value between 0 and 1) which indicates the separation of trajectories; values approaching 1 indicate a clear delineation of the formed trajectories. The model fit log-likelihood (LL) indicates worse model fitness with a larger LL value, and the bootstrap p-value, with non-significant values indicating poor fitness for the tested number of clusters. The proportion of the patients in each class being recommended is more than 5% [7]. The final decision on the number of classes will be determined by a combination of the statistical indices mentioned above and the interpretability and clinical meaning of the trajectories. ***Step 4***, Participants will be assigned to their best-fitting class, and we will use the class memberships as a categorical variable to conduct a multinomial logistic regression model on baseline characteristics. The reference class will be the one including patients with the lowest disability score. We will report the OR with 95% CI and p-value in a table. When the p-value is less than 0.05, we consider the corresponding baseline characteristic as a predictor of a particular trajectory. We will report the missing data proportion and compare drop-out participants' characteristics to those who completed the study[13]. Since statistical models like LCGA assume missing at random (MAR) [9, 13], we will apply full-information maximum likelihood (FIML) estimation to correct the possible bias caused by missing data [13, 17, 18].

We will use the R through *lcmm* [7] package to complete the Latent Class Growth Analysis (LCGA) and multinomial logistic regression.

Figure 1. Spaghetti plot of individual trajectories for each patient

*Results report*

We will report the results including: 1) the number of classes obtained; 2) the trajectory shapes (e.g. linear, quadratic, cubic, etc.); 3) the value of the criteria used to choose the number of trajectories (e.g., BIC) in a table such as Table 1; 4) the baseline characteristics of trajectory membership and the results of multinomial regression analysis such as Table 2; and 5) a figure showing the trajectory subgroups.

**Table 1.** Fit criteria in the LCGA models for choosing the optimal number of classes physical functioning trajectories. N=()

|  | physical functioning trajectories | | | |
| --- | --- | --- | --- | --- |
|  | Model 2 | Mode 3 | Model 4 | …… |
| Model comparison | Model 2 vs model 1 | Model 3 vs model 2 | Model 4 vs model 3 | …… |
| BIC |  |  |  | …… |
| LL |  |  |  | …… |
| bootstrap p-value |  |  |  | …… |
| entropy |  |  |  | …… |
| Patients’ proportion (%) |  |  |  |  |

BIC, Bayesian Information Criterion; LL, log-likelihood; Patients’ proportion (%), the proportion of the patients in the smallest class in a model……

**Table 2**. The baseline characteristics of trajectory membership and the results of multinomial regression analysis

| baseline characteristics | Total Patients (n=, (%)) | Class 1 (n= , (%)) | Class 2 | | | …… |
| --- | --- | --- | --- | --- | --- | --- |
|  |  |  | n= , % | OR (95% CI) | P-value | …… |
| Age (years) |  |  |  |  |  | …… |
| Sex (female) |  |  |  |  |  | …… |
| BMI |  |  |  |  |  | …… |
| …… |  |  |  |  |  | …… |

BMI, Body mass index, ……

**Reference**

1.	Diseases, G.B.D. and C. Injuries, *Global burden of 369 diseases and injuries in 204 countries and territories, 1990-2019: a systematic analysis for the Global Burden of Disease Study 2019.* Lancet, 2020. **396**(10258): p. 1204-1222.

2.	Scheele, J., et al., *Course and prognosis of older back pain patients in general practice: a prospective cohort study.* Pain, 2013. **154**(6): p. 951-7.

3.	da Silva, T., et al., *Recurrence of low back pain is common: a prospective inception cohort study.* J Physiother, 2019. **65**(3): p. 159-165.

4.	Gomes, L.A., et al., *Clinical courses, impact and prognostic indicators for a persistent course of low back pain: Results from a population-based cohort study.* PLoS One, 2023. **18**(3): p. e0265104.

5.	Axen, I. and C. Leboeuf-Yde, *Trajectories of low back pain.* Best Pract Res Clin Rheumatol, 2013. **27**(5): p. 601-12.

6.	Nagin, D.S., *Group-Based Modeling of Development*. 2005: Harvard University Press.

7.	Nguena Nguefack, H.L., et al., *Trajectory Modelling Techniques Useful to Epidemiological Research: A Comparative Narrative Review of Approaches.* Clin Epidemiol, 2020. **12**: p. 1205-1222.

8.	Gatchel, R.J., et al., *Transitioning from Acute to Chronic Pain: An Examination of Different Trajectories of Low-Back Pain.* Healthcare (Basel), 2018. **6**(2).

9.	Silva, J.P.D., et al., *Trajectories of pain and disability in older adults with acute low back pain: Longitudinal data of the BACE-Brazil cohort.* Braz J Phys Ther, 2022. **26**(1): p. 100386.

10.	Deyo, R.A., et al., *Trajectories of symptoms and function in older adults with low back disorders.* Spine (Phila Pa 1976), 2015. **40**(17): p. 1352-62.

11.	Enthoven, W.T., et al., *Defining trajectories in older adults with back pain presenting in general practice.* Age Ageing, 2016. **45**(6): p. 878-883.

12.	Wong, A.Y., et al., *Global Consensus From Clinicians Regarding Low Back Pain Outcome Indicators for Older Adults: Pairwise Wiki Survey Using Crowdsourcing.* JMIR Rehabil Assist Technol, 2019. **6**(1): p. e11127.

13.	van de Schoot, R., et al., *The GRoLTS-Checklist: Guidelines for Reporting on Latent Trajectory Studies.* Structural Equation Modeling: A Multidisciplinary Journal, 2017. **24**(3): p. 451-467.

14.	Scheele, J., et al., *Back complaints in the elders (BACE); design of cohort studies in primary care: an international consortium.* BMC Musculoskelet Disord, 2011. **12**: p. 193.

15.	Roland, M. and R. Morris, *A study of the natural history of back pain. Part I: development of a reliable and sensitive measure of disability in low-back pain.* Spine (Phila Pa 1976), 1983. **8**(2): p. 141-4.

16.	Jung, T. and K.A.S. Wickrama, *An Introduction to Latent Class Growth Analysis and Growth Mixture Modeling.* Social and Personality Psychology Compass, 2008. **2**(1): p. 302-317.

17.	Ray, J.V., et al., *The Impact of Missing Risk Factor Data on Semiparametric Group-Based Trajectory Models.* Journal of Developmental and Life-Course Criminology, 2018. **4**(3): p. 276-296.

18.	Nagin, D.S. and C.L. Odgers, *Group-based trajectory modeling in clinical research.* Annu Rev Clin Psychol, 2010. **6**: p. 109-38.
